# Supplementary material for: Clinical, anamnestic, and sociodemographic predictors of positive SARS-CoV-2 testing in children: A cross sectional study in a tertiary hospital in Italy
Source: PLoS One. 2022 Jan 25;17(1):e0262923. doi: 10.1371/journal.pone.0262923 (PMC8789147; doi:10.1371/journal.pone.0262923)
Supplement: Attachment — Submitted filename: PONE-D-21-24181 Response to reviewers.docx [file pone.0262923.s002.docx]

PONE-D-21-24181
Clinical, anamnestic, and sociodemographic predictors of positive SARS-CoV-2 testing in children: a cross sectional study in a tertiary hospital in Italy

Review Comments to the Author

Please use the space provided to explain your answers to the questions above. You may also include additional comments for the author, including concerns about dual publication, research ethics, or publication ethics. (Please upload your review as an attachment if it exceeds 20,000 characters)

**Reviewer #1**: The study is well written and the analyses well performed.

***Thank you for your appreciation.

However, there is a significant basic mistake that does not allow to give much significance to this study.
In particular, a single swab performed in the emergency department without information on what happened after discharge has really no sense to me.

***Thank you for your comment. At that time, the general process after a positive swab envisaged the activation of quarantine and a swab at two weeks by the Department of Prevention. Thus, a follow up (control) swab was always performed, but these follow up swabs could have been performed in other health services, not necessary in our Institute. Moreover, the aim of this study was to identify clinical, anamnestic, and sociodemographic characteristics associated with a positive swab for SARS-CoV-2 in a population of children, hence we aimed at investigating a real life, pragmatic approach in order to try to add some clinical hints for clinicians.

Additionally, the swabs were performed in an outpatient clinic, as mentioned in the manuscript, not in an emergency department, and we aimed at investigating the symptoms presented at the time of the swab to observe if specific symptoms could be predictive of testing positivity, thus the follow up swab would have added, in our opinion, not significant information to the aim of the study.

How can you exclude that you had a false positive result? For example, more than 200 children from the "negative" group had known contacts. Also, a number of children from the negative group had anosmia and ageusia, which you itself define as a predictive symptoms. Importantly, these symptoms are very unusual in other diseases.
As a proof that the study, although well written and analysed, has low significance, you define that a very common and usual presentation of children in the ED can be predictive of covid. However, we know that so many children can have mild or asymptomatic disease.

***Thank you for your comment. The sensitivity and specificity of the RT-PCR nasopharyngeal swab to detect SARS-CoV-2 declared by the manufacturer are very high (about 100%), so the possibility to have a false positive or negative can be considered pretty low, as also showed in the literature (1).

Moreover, regarding the false positives, the swab and kits used for the analysis are certified to identify 3 different genes for all variants that are circulating to date. For each swab the quantity and quality of the material taken was checked, controlling that it was sufficient to highlight up to 5 copies of the virus (lower limit of the test). In case of a doubtful result (eg 2 out of 3 genes positive) the patient was asked to perform again the swab, thus the possibility to have false positives/negatives is very remote. This further information has been included in the manuscript in the section Laboratory methods.

About the lost among the cases tested negative possible positive SARS-CoV-2 might be realistic, we observed the trend on another study we conducted on serology, but the aim of this paper was not to demonstrate the circulation of the virus.

Furthermore, we do agree that the child can have been asymptomatic or mild, but firstly at that time of the pandemic all children also very slight symptoms were tested, and secondly this study explicitly included only children with symptoms to provide a predictive score to identify children at risk of COVID-19, we were not aiming at identifying all children with a positivity to SARS-CoV-2.

We also would like to thank you also for the comment on anosmia and ageusia. Although the symptoms are quite specific, those are not exclusive of COVID-19, but they might have other causes (such as allergies, other viral upper respiratory tract infections, seasonality) (2-3). Thus, those negatives presenting anosmia or ageusia might not be per se false negatives. Furthermore, also other studies reported anosmia and ageusia both in positive and negative cases, but they are more common among positives (3-5), this information has been inserted in the discussion.

Finally, as we mentioned in our limits families were directly providing information, and some symptoms might have been misinterpreted, and anosmia and ageusia might be one of these considering the difficulty in detecting such symptoms in children.

1. Floriano I, Silvinato A, Bernardo WM, Reis JC, Soledade G. Accuracy of the Polymerase Chain Reaction (PCR) test in the diagnosis of acute respiratory syndrome due to coronavirus: a systematic review and meta-analysis. Rev Assoc Med Bras (1992). 2020 Jul;66(7):880-888. doi: 10.1590/1806-9282.66.7.880. Epub 2020 Aug 24. PMID: 32844930.
2. Pierron D, Pereda-Loth V, Mantel M, Moranges M, Bignon E, Alva O, Kabous J, Heiske M, Pacalon J, David R, Dinnella C, Spinelli S, Monteleone E, Farruggia MC, Cooper KW, Sell EA, Thomas-Danguin T, Bakke AJ, Parma V, Hayes JE, Letellier T, Ferdenzi C, Golebiowski J, Bensafi M. Smell and taste changes are early indicators of the COVID-19 pandemic and political decision effectiveness. Nat Commun. 2020 Oct 14;11(1):5152. doi: 10.1038/s41467-020-18963-y. PMID: 33056983; PMCID: PMC7560893.
3. Haehner A, Draf J, Dräger S, de With K, Hummel T. Predictive Value of Sudden Olfactory Loss in the Diagnosis of COVID-19. ORL J Otorhinolaryngol Relat Spec. 2020;82(4):175-180. doi: 10.1159/000509143. Epub 2020 Jun 11. PMID: 32526759; PMCID: PMC7360503.
4. Sudre CH, Keshet A, Graham MS, Joshi AD, Shilo S, Rossman H, Murray B, Molteni E, Klaser K, Canas LD, Antonelli M, Nguyen LH, Drew DA, Modat M, Pujol JC, Ganesh S, Wolf J, Meir T, Chan AT, Steves CJ, Spector TD, Brownstein JS, Segal E, Ourselin S, Astley CM. Anosmia, ageusia, and other COVID-19-like symptoms in association with a positive SARS-CoV-2 test, across six national digital surveillance platforms: an observational study. Lancet Digit Health. 2021 Sep;3(9):e577-e586. doi: 10.1016/S2589-7500(21)00115-1. Epub 2021 Jul 22. Erratum in: Lancet Digit Health. 2021 Sep;3(9):e542. PMID: 34305035; PMCID: PMC8297994.
5. Mak PQ, Chung KS, Wong JS, Shek CC, Kwan MY. Anosmia and Ageusia: Not an Uncommon Presentation of COVID-19 Infection in Children and Adolescents. Pediatr Infect Dis J. 2020 Aug;39(8):e199-e200. doi: 10.1097/INF.0000000000002718. PMID: 32516281.

I am very very sorry to see that you did a huge effort in this analyses, but really the hypothesis behind the design of this study has a very doubtful significance. You should use these data to make a new one, doing f-up swabs as a minimum, or interview of parents to understand if further swabs have been done and the results to see how many of them are true negative. For example, a preprint from UK recently found that 1 out of 3 students had IgG positive (!!) confirming that the virus circulates much more than expected. And I am quite sure that among the negative, particularly those with known contacts and with anosmia/ageusia, you missed several cases.

***Thank you for your comment. We acknowledge the fact that the high rate of asymptomatic children might limit the predictiveness of symptoms, but we believe that this is an independent variable from the false positive issue.

Moreover, IgG testing measures positivity in the span of several months, so that in a methodologic perspective, could not be related to symptoms or swab positivity in a defined moment. Finally, as mentioned above, the aim of this paper was not to demonstrate the circulation of the virus, but to identify clinical, anamnestic, and sociodemographic characteristics associated with a positive swab for SARS-CoV-2 in a population of children.

**Reviewer #2**: In the manuscript authored by Benedetta Armocida et al, authors summarize clinical, anamnestic, and sociodemographic characteristics of children affected or not by Sars Cov-2 in a frame period between September 20th and December 23rd 2020.
A major concern is that authors provided a study that is more descriptive then predictive, as stated from authors. This is a critical point.

***Thank you for your comment. We acknowledge the fact that the high rate of asymptomatic children makes limits the predictiveness of symptoms, but we believe that this is an independent variable from the false positive issue.

We agree that in a blurred clinical setting, as SARS-CoV-2 infection in children, every study based on symptoms carries the risk of being more descriptive than predictive. This matter has been specified in text as a limitation, although concrete data on these issues are scant.

No follow up is present.

***Thank you for your comment. At that time the general process after a positive swab envisaged the activation of quarantine and a swab at two weeks by the department of prevention. Thus, a follow up (control) swab was always performed, but these follow up swabs could have been performed in other departments or hospitals, not necessary in our Institute. Moreover, the aim of this study was to identify clinical, anamnestic, and sociodemographic characteristics associated with a positive swab for SARS-CoV-2in a population of children, hence we aimed at investigating a real life, pragmatic approach in order to try to add some clinical hints for clinicians and the symptoms presented at the time of the swab to observe if specific symptoms could be predictive of testing positivity, thus the follow up swab would have added, in our opinion, not significant information to the aim of the study.

ROC curve is computed between 127 positive vs 1357 negative. Are these groups comparable?

***Thank for your comment. The two groups are comparable. As mentioned in the study population and in the inclusion criteria, all cases were children (2-14 years of age), coming from the general population of Trieste, all attending school or education services, and turning to the territorial services to undergo a RT-PCR nasopharyngeal swab to detect SARS-CoV-2, and only the ones with symptoms were included in the study.

It is not clear to the reader if authors suggest NOT to perform Sars-Cov2 diagnostic test in children presenting rhinorrhoea, sore throat, or diarrhoea.

***Thank for your comment. To clarify, we are not suggesting clinicians not to perform SARS-CoV-2 diagnostic test in children presenting rhinorrhea, sore throat, or diarrhea, although we add the information that these symptoms carry a low risk of SARS-COV 2 infection. The information might be very relevant and might be helpful in orienting choices in specific settings, such as these with limited resources. This information has been included in the Discussion
