# Supplementary material for: Clinical, anamnestic, and sociodemographic predictors of positive SARS-CoV-2 testing in children: A cross sectional study in a tertiary hospital in Italy
Source: PLoS One. 2022 Jan 25;17(1):e0262923. doi: 10.1371/journal.pone.0262923 (PMC8789147; doi:10.1371/journal.pone.0262923)
Supplement: Attachment — Submitted filename: PONE-D-21-24181 Response to reviewers.docx [file pone.0262923.s003.docx]

PONE-D-21-24181R1

Clinical, anamnestic, and sociodemographic predictors of positive SARS-CoV-2 testing in children: a cross sectional study in a tertiary hospital in Italy

PLOS ONE

Review Comments to the Author

Please use the space provided to explain your answers to the questions above. You may also include additional comments for the author, including concerns about dual publication, research ethics, or publication ethics. (Please upload your review as an attachment if it exceeds 20,000 characters)

Reviewer #1: I still think that a very single test cannot fully exclude covid in the negative control group, and therefore a study aiming to detect positive cases at the time of testing, without knowing days of illness and further follow-up is of limited value for a prestigious journal

***Thank you for your comment. We acknowledge and agree that a single test cannot completely exclude the positives in the negatives control group, therefore we explicitly included this point as limitations of the study. However, we considered this possibility extremely small, as our Institute was the only hub performing COVID-19 swab in the Trieste area (information added at the beginning of the methods section), hence in case of persistent or worsening symptoms, the access of these children for a second swab would have been performed within our Institute and this didn’t happen from our available data.

Reviewer #2: Authors provided most of the corrections requested from the reviewers and now the manuscript has been higly improved.

***Thank you for your appreciation.
