# Supplementary material for: Clinical, anamnestic, and sociodemographic predictors of positive SARS-CoV-2 testing in children: A cross sectional study in a tertiary hospital in Italy
Source: PLoS One. 2022 Jan 25;17(1):e0262923. doi: 10.1371/journal.pone.0262923 (PMC8789147; doi:10.1371/journal.pone.0262923)
Supplement: Attachment — Submitted filename: PONE-D-21-24181 Response to reviewers.docx [file pone.0262923.s004.docx]

PONE-D-21-24181R2

Clinical, anamnestic, and sociodemographic predictors of positive SARS-CoV-2 testing in children: a cross sectional study in a tertiary hospital in Italy

PLOS ONE

Reviewer #1: thank you very much for this clarification, and thank you for having included it in the limitation section. this is an helpful addition and I think also you will benefit from this clarification when other colleagues will read this paper.
I only ask one little addition:
the authors NEVER compared in the discussion their findings with other italian ones, this should be done and particularly with an early national italian report , to also address if symptoms presentation have changed after the very early months (please refer to COVID-19 in 17 Italian Pediatric Emergency Departments, Parri, N., et al, Pediatrics, 2020, 146(6), e20201235) and even other ones would be appreciated

***Thank you very much for this additional request. We have included two Italian studies as references and compared the main findings also with an Italian study carried out in a similar outpatient setting and in the same school aged population (reference 8).

References added:

Parri N, Lenge M, Cantoni B, Arrighini A, Romanengo M, Urbino A, et al. COVID-19 in 17 Italian Pediatric Emergency Departments. Pediatrics. 2020 Dec;146(6):e20201235. doi: 10.1542/peds.2020-1235. Epub 2020 Sep 23. PMID: 32968031.

Lazzerini M, Sforzi I, Trapani S, Biban P, Silvagni D, Villa G, et al. COVID-19 Italian Pediatric Study Network. Characteristics and risk factors for SARS-CoV-2 in children tested in the early phase of the pandemic: a cross-sectional study, Italy, 23 February to 24 May 2020. Euro Surveill. 2021 Apr;26(14):2001248. doi: 10.2807/1560-7917.ES.2021.26.14.2001248. PMID: 33834960; PMCID: PMC8034058.

As mentioned in the first paragraph of our discussion though, to our knowledge, this is the first study to provide a predictive score for the paediatric population to identify children at risk to be positive to the SARS-CoV-2 test.

Reviewer #3: This is an interesting study performed by Armocida et al, investigating the clinical, anamnestic and sociodemographic factors at the time of a positive SARS-CoV-2 test in children. The authors constructed a predictive model of SARS-CoV-2 positivity.

My only concern is that whether any of the negative patients subsequently developed SARS-CoV-2. The authors state that none of the negative patients tested positive within their laboratory. Do they have data on whether children were retested and gave a negative test or there simply were no positive tests? My suspicion would be that if a child was negative then the likelihood of being retested would be relatively low. Without systematically testing patients while they continued to have symptoms then it is difficult to definitively answer this question. The authors must make it clear that these are the symptoms that the children had whilst they tested positive.

***Thank you very much for this comment. Unfortunately, we don’t have this additional information. As we mentioned in the limits, we acknowledge the possible limit in performing only one single test, which, without a further systematic follow-up, might have not detected a child positive to SARS-CoV-2, who might have been included in the negative control group. However, we considered this possibility extremely small, as our Institute was the only hub performing COVID-19 swab in the Trieste area, hence in case of persistent or worsening symptoms, the access of these children for a second swab would have been performed within our Institute and this didn’t happen from our available data.

We added a sentence in the Methods section to clarify that we collected information on specific symptoms that led to the swab being administered.

In addition, do the authors have any data on how long the symptoms persisted prior to a positive test?

***Thank you very much for this comment. Unfortunately, we don’t have this additional information as data were collected at the time the RT-PCR testing was performed. This information was reported in Methods “data collection” section.
